# Supplementary material for: Technological Properties of Inulin-Enriched Doughs and Breads, Influence on Short-Term Storage and Glycemic Response
Source: Foods. 2024 Aug 27;13(17):2711. doi: 10.3390/foods13172711 (PMC11395101; doi:10.3390/foods13172711)
Supplement: Supplementary file 1 [file foods-13-02711-s001.zip › foods-3170519-supplementary.pdf]

Supplemental tables

Table S1. Principal Component Analysis (PCA). Eigenvalue and proportion of variance explained by each principal component.

PC	Eigenvalue	Variance (%)	Cumulative (%)
1	29,1758	64,835	64,835
2	12,4585	27,686	**92,521**
3	3,3657	7,4793	

Table S2. Principal component analysis (PCA) loadings of the physical and chemical characteristics of flours, doughs, and breads with different inulin integration.

	Axis 1	Axis 2	Axis 3	Axis 4	Axis 5
WB_(g/g_H2O)	-0,9723	0,2329	-0,02137	-2,79E-16	1,11E-32
OB_(g/g_oil)	0,1958	0,8642	-0,4634	-4,89E-15	-3,31E-34
Mixing_Time_(min)	0,9445	-0,2169	-0,2465	-3,61E-16	3,79E-34
Peak_dough_height_(M.U)	0,5153	-0,4326	0,7398	1,72E-16	3,53E-34
Dough_development_time_(min)	0,9323	0,2628	-0,2483	-2,42E-16	2,75E-34
Stability_(min)	0,2596	0,7873	0,5592	3,37E-16	-1,64E-35
Water_absorption_at_500_B.U_(%)	-0,9678	0,1212	0,2207	-2,55E-16	-3,93E-34
W_(Jx10-4)	0,7815	-0,6009	0,1678	1,19E-16	4,48E-34
P/L	0,944	-0,2709	-0,1885	1,51E-16	4,14E-34
Brown_index_(100-L)	0,8513	-0,5222	0,05102	1,11E-17	4,45E-34
a*	0,9528	-0,2969	-0,06407	1,16E-16	4,31E-34
b*	0,7297	0,5788	-0,364	7,21E-16	1,57E-34
Brown_index_(100-L)	-0,8961	0,4423	0,03716	-5,87E-16	-4,60E-34
a*	0,7131	-0,634	-0,2992	1,68E-16	3,96E-34
b*	0,4926	0,8668	-0,07679	-8,47E-16	-3,53E-35
Moisture_T0	-0,8228	0,5385	0,1818	-4,88E-16	-4,39E-34
Moisture_T2	-0,6859	0,6191	0,3825	-1,35E-15	-4,21E-34
Moisture_T4	-0,5588	0,7482	0,3578	-8,48E-16	-3,83E-34
VOLUME_T0	-0,9854	-0,1426	0,09298	1,90E-16	-3,35E-34
VOLUME_T2	-0,9698	-0,2087	0,1259	2,17E-16	-3,11E-34
VOLUME_T4	-0,9919	-0,1222	-0,03453	1,77E-16	-3,52E-34
Height_T0	-0,9663	-0,1972	-0,1655	5,90E-16	-3,20E-34
Height_T2	-0,9054	-0,3501	-0,2403	-6,06E-17	-2,93E-34
Height_T4	-0,9058	-0,3561	-0,2295	5,06E-16	-2,70E-34
Weight_T0	0,9423	-0,3165	-0,1093	-6,66E-17	4,21E-34
Weight_T2	0,9797	-0,194	-0,0505	-1,21E-16	4,11E-34
Weight_T4	0,932	-0,2823	-0,2274	-1,85E-15	3,33E-34
Porosity_T0	0,9168	-0,3264	0,2301	2,23E-16	4,49E-34
Porosity_T2	0,9168	-0,3264	0,2301	2,23E-16	4,49E-34
Porosity_T4	0,993	-0,1142	0,02852	-3,44E-16	3,96E-34
Hardness_T0	0,4525	-0,7763	0,4388	4,91E-16	3,93E-34
Hardness_T2	-0,6548	-0,7502	-0,09181	9,49E-16	-6,09E-35

Hardness_T4	-0,8253	-0,108	0,5543	1,52E-16	-2,48E-34
Springiness_T0	0,2795	0,9321	-0,2305	6,97E-15	1,54E-34
Springiness_T2	0,09186	0,994	-0,05933	9,26E-16	-1,49E-34
Springiness_T4	0,6705	0,7364	0,09019	1,88E-17	1,07E-34
Gumminess_T0	0,8911	0,3629	0,2725	-4,79E-16	2,67E-34
Gumminess_T2	0,9959	-0,0171	-0,08852	4,49E-17	3,83E-34
Gumminess_T4	0,6344	0,7488	0,1919	-2,67E-16	8,66E-35
Chewiness_T0	0,8876	0,3694	0,2751	2,99E-17	2,84E-34
Chewiness_T2	0,8446	-0,1252	0,5206	-4,86E-17	3,89E-34
Chewiness_T4	0,6239	0,7376	0,2584	-2,95E-16	8,88E-35
Cohesivness_T0	-0,8869	0,4116	-0,2099	-4,00E-17	-4,48E-34
Cohesivness_T2	0,4911	0,8612	-0,1311	9,94E-16	3,11E-35
Cohesivness_T4	0,8188	0,5659	-0,09636	4,49E-18	1,86E-34
